# Activated Oncogenic Pathway Modifies Iron Network in Breast Epithelial Cells: A Dynamic Modeling Perspective

**DOI:** 10.1371/journal.pcbi.1005352

**Published:** 2017-02-06

**Authors:** Julia Chifman, Seda Arat, Zhiyong Deng, Erica Lemler, James C. Pino, Leonard A. Harris, Michael A. Kochen, Carlos F. Lopez, Steven A. Akman, Frank M. Torti, Suzy V. Torti, Reinhard Laubenbacher

**Affiliations:** 1 Department of Mathematics and Statistics, American University, Washington, DC, USA; 2 The Jackson Laboratory, Bar Harbor, ME, USA; 3 Department of Molecular Biology and Biophysics, University of Connecticut Health Center, Farmington, CT, USA; 4 Chemical and Physical Biology Graduate Program, Vanderbilt University, Nashville, TN, USA; 5 Department of Cancer Biology, Vanderbilt University, Nashville, TN, USA; 6 Department of Biomedical Informatics, Vanderbilt University, Nashville, TN, USA; 7 Center for Quantitative Science, Vanderbilt University, Nashville, TN, USA; 8 Cancer Program, Roper St Francis HealthCare, Charleston, SC, USA; 9 Department of Medicine, University of Connecticut Health Center, Farmington, CT, USA; 10 Center for Quantitative Medicine, University of Connecticut Health Center, Farmington, CT, USA; 11 The Jackson Laboratory for Genomic Medicine, Farmington, CT, USA; Northeastern University, UNITED STATES

## Abstract

Dysregulation of iron metabolism in cancer is well documented and it has been suggested that there is interdependence between excess iron and increased cancer incidence and progression. In an effort to better understand the linkages between iron metabolism and breast cancer, a predictive mathematical model of an expanded iron homeostasis pathway was constructed that includes species involved in iron utilization, oxidative stress response and oncogenic pathways. The model leads to three predictions. The first is that overexpression of iron regulatory protein 2 (IRP2) recapitulates many aspects of the alterations in free iron and iron-related proteins in cancer cells without affecting the oxidative stress response or the oncogenic pathways included in the model. This prediction was validated by experimentation. The second prediction is that iron-related proteins are dramatically affected by mitochondrial ferritin overexpression. This prediction was validated by results in the pertinent literature not used for model construction. The third prediction is that oncogenic Ras pathways contribute to altered iron homeostasis in cancer cells. This prediction was validated by a combination of simulation experiments of Ras overexpression and catalase knockout in conjunction with the literature. The model successfully captures key aspects of iron metabolism in breast cancer cells and provides a framework upon which more detailed models can be built.

## Author Summary

Iron is required for cellular metabolism and growth, but can be toxic due to its ability to cause high oxidative stress and consequently DNA damage. To prevent damage, all organisms that require iron have developed mechanisms to tightly control iron levels. Dysregulation of iron metabolism is detrimental and can contribute to a wide range of diseases, including cancer. This paper presents a predictive mathematical model of iron regulation linked to iron utilization, oxidative stress, and the oncogenic response specific to normal breast epithelial cells. The model uses a discrete modeling framework to generate novel biological hypotheses for an investigation of how normal breast cells become malignant cells, capturing a breast cancer phenotype of iron homeostasis through overexpression and knockout simulations. The new biology discovered is (1) IRP2 overexpression alters the iron homeostasis pathway in breast cells, without affecting the oxidative stress response or oncogenic pathways, (2) an activated oncogenic pathway disrupts iron regulation in breast cancer cells.

## Introduction

Every aerobic organism requires iron for energy production, DNA synthesis, oxygen transport, and cellular respiration. However, this essential element has the potential to exist in various oxidation states and can enable the formation of reactive oxygen species. To avoid iron toxicity, all organisms requiring iron have developed a complex machinery to tightly control iron at both the systemic and the cellular levels. Our goal here is to understand how in cancer this machinery is altered. Dysregulation of iron metabolism in cancer is well documented, and it has been suggested that there is interdependence between excess iron and increased cancer incidence and progression [1]. Recently, it was observed that reduced levels of ferroportin, a cellular iron exporter, were associated with poor clinical outcome [2]. In the same study, a direct relationship between intracellular iron and tumor growth was demonstrated, and in subsequent work it was shown that high expression levels of the major iron importer, transferrin receptor 1, and reduced levels of the gene HFE, were also associated with poor prognosis in breast cancer patients [3].

In a previous study, we constructed a dynamic mathematical model of the core iron homeostasis control system in normal breast epithelial cells [4]. This choice of cell type was motivated by our interest in the role of intracellular iron homeostasis in the pathogenesis of breast cancer. For the core control system we have focused on the proteins responsible for iron import, export, and sequestration, together with the iron regulatory proteins and the labile iron pool. We validated the model using experimental data from overexpression of ferroportin. Our analytical arguments and extensive simulations demonstrated that the model reaches a unique stable steady state for any choice of parameters, agreeing with experimental evidence that cellular iron is tightly controlled [5].

We hypothesized there that major signaling pathways activated in cancer disrupt this iron regulatory network. To test this hypothesis, it was necessary to first connect the core iron network to known molecules whose expression levels are altered in cancer. Here, we build and analyze an intracellular mathematical model specific to normal breast epithelial cells that dynamically links iron metabolism to species from iron utilization, the oxidative stress response, and oncogenic pathways. The model has allowed us to highlight dynamical features of the system and identify key players in the system that lead to different phenotypes without having to perform lengthy laboratory experiments. We have validated the model using experimental data and literature, and confirmed that the iron homeostasis pathway can be modified by activating an oncogenic pathway.

Several models related to iron homeostasis have been developed and analyzed. Some are aimed at systemic iron homeostasis, consisting of a number of compartments that capture the amount of iron at a particular location [6–10]. Other models are cell type specific (kidney) [11] or organ specific (liver) [12]. One of the first intracellular models of iron metabolism was proposed by Omholt et al. [13], but this model did not explicitly include proteins responsible for iron export and sequestration. Our earlier model [4] included this additional feedback structure and was further considered by Mobilia et al. [14], where the authors concentrated on the same five species but a different system of differential equations. While all of these models are valuable and address specific questions, none of of them, including our earlier model, connect the iron network to an oncogenic pathway. The approach of identifying and uniting different biochemical pathways was previously explored by Funke et al. [15], where the authors attempted to explain Parkinson’s disease by considering gene products involved in disease, and also included iron. We took a similar approach by producing one coherent model integrating several pathways connected to the iron network. Our new model has a potential for further inclusion of other pathways to produce a more comprehensive picture of dysregulation of iron metabolism in cancer.

### The Iron Core Control System and Expanded Network

This section provides biological background about iron metabolism and its connection to some oncogenic pathways.

#### Iron metabolism

Free ferrous iron contributes to the formation of hydroxyl radicals through the Fenton reaction, so intracellular iron levels are meticulously maintained in order to limit toxicity. Iron levels are controlled by iron-regulatory proteins (IRPs) that coordinate intracellular iron uptake, utilization, storage, and excretion. What follows is a brief description of the core iron control system. For an overview of intracellular and systemic iron homeostasis see [16].

Ferric iron, Fe^3+^, circulates in plasma bound to transferrin (Tf), a glycoprotein with two binding sites for ferric iron. Tf retains iron in a soluble form, which limits the formation of toxic radicals, and delivers iron to cells, with uptake mediated predominantly by transferrin receptor 1 (TfR1). Iron-loaded Tf (Holo-Tf) is taken up by receptor-mediated endocytosis into acidified endosomes where ferric iron is reduced to ferrous iron, Fe^2+^, with the assistance of STEAP proteins. From the endosomes, Fe^2+^ is transported into the cytoplasm via divalent metal transporter 1 (DMT1). We note that, in some cells, DMT1 is also located on the cell surface and participates in the transport of extracellular iron. However, the role of DMT1 in peripheral tissues is less studied. Thus, in our model we only consider TfR1 as the major iron importer. From the endosomes, iron enters the labile iron pool (LIP), a cytosolic pool of weakly bound iron. Ferroportin (Fpn), located on the plasma membrane, is believed to be the only ferrous iron exporter. Excess ferrous iron that is not exported or utilized is oxidized by the cytosolic protein ferritin (Ft), and is sequestered into its ferrihydrite mineral core.

The iron regulatory proteins IRP1 and IRP2 regulate iron homeostasis post-transcriptionally by binding to iron responsive elements (IREs), cis-regulatory hairpin structures that are present in the untranslated regions (UTRs) of mRNAs involved in iron metabolism. The mRNAs encoding ferritin and ferroportin contain a single IRE in their 5’UTRs. The mRNA encoding TfR1 contains multiple IREs within the 3’UTR. In iron-deplete cells, IRPs are active and have high affinity for IREs. Binding to the 3’UTR IREs results in the stabilization of mRNA of TfR1, and binding of IRPs to the single 5’UTR IRE inhibits the translation of ferroportin and ferritin. In iron-replete conditions, IRPs have reduced affinity for IREs and their regulatory effect is attenuated, which results in the degradation of TfR1 mRNA and translation of ferroportin and ferritin mRNAs. In the case of IRP1, the reduction in affinity for IREs results from a completed iron sulfur cluster that impedes IRE binding; in the case of IRP2, loss of binding to the IRE is due to ubiquitination and degradation of IRP2.

The peptide hormone hepcidin (Hep) regulates systemic iron homeostasis by inhibiting iron release from duodenal enterocytes, macrophages, and hepatocytes. Hepcidin binds to the iron exporter ferroportin and triggers its internalization and degradation in lysosomes. Hep is transcriptionally induced by bone morphogenetic proteins (BMPs) and the inflammatory cytokine interleukin-6 (IL-6). The induction of hepcidin by IL-6 is thought to be a major contributor to the hypoferremia that frequently accompanies chronic infections, acute inflammation and cancer [17]. Recently, it has been established that breast epithelial cells also express hepcidin and that it plays an important role in peripheral tissue by regulating ferroportin [2].

#### Iron utilization

The mitochondria are the major site of iron utilization. Cytosolic iron is imported into the mitochondria by the SLC transporter mitoferrin (Mfrn), to be incorporated into protoporphyrin IX (PPIX) during heme synthesis. There are two homologs, mitoferrin-1 (SLC25A37), which is expressed at high levels in erythroblasts and at low levels in other tissue, and mitoferrin-2 (SLC25A28), which is expressed ubiquitously [18]. Once iron is transported into the mitochondria it is then used in heme synthesis, iron sulfur cluster (ISC) synthesis, or enters mitochondrial ferritin (Ftmt). Just like cytosolic ferritin, Ftmt is an iron storage protein. It is encoded by an intronless gene located on chromosome 5q23.1, but lacks a consensus IRE sequence [19, 20]. The primary function of Ftmt is not fully understood, but evidence indicates that its role is to protect mitochondria from iron-dependent oxidative damage [21]. We will denote the mitochondrial labile iron pool by LIPmt.

It is well established that intracellular heme regulates its own production and degradation through delta aminolevulinate synthase (ALAS) and heme oxygenase (HO), respectively. There are three distinct isozymes of HO: HO-1 (inducible form), and HO-2 and HO-3 (constitutive forms) [22, 23]. We include the inducible isoform HO-1 in our mathematical model because the expression of HO-1 is altered by oxidative stress, and because HO-1 contributes to tumorigenicity in many cancers [24–28]. HO-1 maintains heme homeostasis by initiating the oxidative cleavage of heme to ferrous iron (Fe^2+^), carbon monoxide (CO), and biliverdin [29]. Moreover, HO-1 inhibits the expression of IL-6, thus also taking on an anti-inflammatory function [30]. ALA synthase has two forms: ALAS1, which is ubiquitously expressed and is downregulated by heme, and ALAS2, which is erythroid-specific, and is regulated by the IRP system [31–33]. Since our model is tissue specific, our network only includes ALAS1. Heme synthesis involves several steps that occur in two compartments: (i) mitochondria with the initial and final steps, and (ii) cytosol with intermediate steps. The ALA synthase reaction is the committed step of heme synthesis. Heme negatively regulates ALAS by multiple feedback mechanisms, including effects on transcription, mRNA stability, and mitochondrial translocation of ALAS [34]. The mitochondria export the product, *δ*-ALA, to the cytoplasm, where the next four reactions occur. The final steps of heme synthesis occur in the mitochondria, where Fe^2+^ is incorporated into PPIX via ferrochelatase, which completes heme synthesis [33, 35, 36].

**Fig 1:**
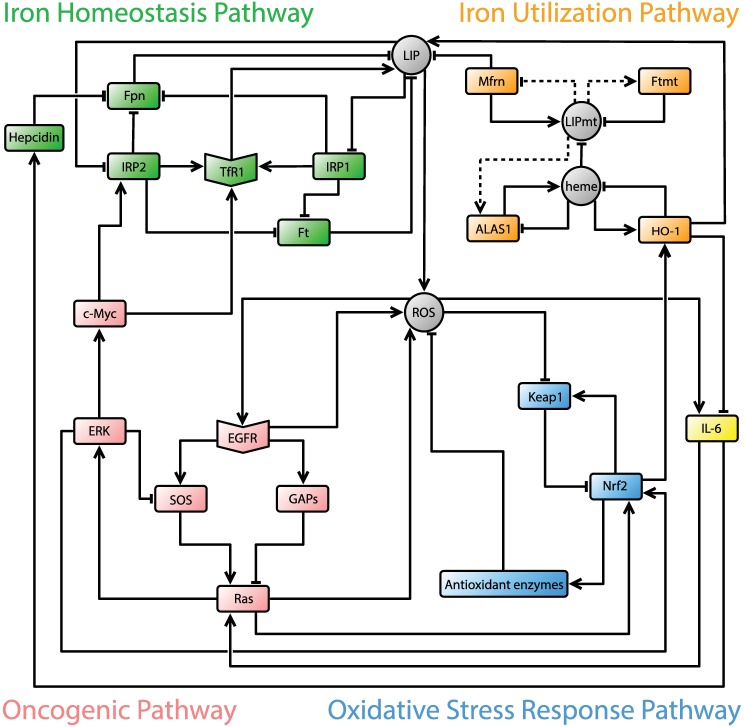
Intracellular iron network. Iron homeostasis pathway depicted in green (LIP, TfR1, Fpn, Ft, IRP1, IRP2, Hep), iron utilization depicted in orange (Mfrn, LIPmt, Ftmt, ALAS1, heme, HO-1), oxidative stress response depicted in blue (ROS, Keap1, Nfr2, Antioxidant enzymes), and oncogenic pathway depicted in pink (EGFR, SOS, GAPs, Ras, ERK, c-Myc). IL-6, in yellow, is the only inflammatory cytokine in the network. Arrows represent activation/upregulation and hammer heads represent inhibition/downregulation. Dashed connections are explained in the iron utilization subsection of the introduction. Rectangular shapes represent proteins/enzymes, circular; molecules, hexagon-like; receptors. CellDesigner [67] was used for visualization.

We do not include all the intermediate players involved in heme biosynthesis since all reactions occur in sequence, and, from a mathematical standpoint, this will not affect the dynamic behavior of the model. On the other hand, in iron-deplete conditions, heme synthesis will not be completed and thus we will assume a feedback regulation from LIPmt to ALAS1 to allow for this possibility (represented in our model by a dotted arrow in Fig 1). In addition, although our understanding of the precise regulation of Ftmt and Mfrn is incomplete, experimental evidence suggests that a feedback mechanism must exist, which responds to the levels of LIPmt, and some authors suggest that there might be even cross-talk between cytosolic and mitochondrial iron metabolism [21, 37]. The dotted arrows in Fig 1 represent the feedback mechanism from LIPmt to Ftmt and Mfrn. Our current model does not include iron-sulfur cluster (ISC) synthesis due to the complexity and incompletely understood nature of this process in mammalian cells [38].

#### Oxidative stress

Oxidative stress is an imbalance between the production of reactive oxygen species (ROS) by oxygen-dependent metabolic reactions and the production of antioxidants. Oxidative stress can result from excess production of ROS, insufficient production of cytoprotective proteins that produce antioxidants and detoxify ROS (here termed antioxidant enzymes), or a combination of both. Reactive oxygen species are a family of molecules with one or more unpaired electrons, and are generated during many cellular processes. Antioxidant enzymes, such as superoxide dismutase, catalase, and glutathione peroxidase, defend the organism by neutralizing free radicals and thus protecting cells from oxidative stress and oxidative damage to DNA, lipids, and proteins [39].

Iron can contribute to the formation of ROS. In aerobic organisms, oxygen (O_2_) is mostly bound to hydrogen (H_2_) as water. However, a small portion of O_2_ can be converted to a variety of reactive oxygen species (ROS), including the superoxide radical (O_2_·)^−^, hydrogen peroxide H_2_O_2_ and the hydroxyl radical ·OH [40, 41]. Ferrous iron *Fe*^2+^ can interact with O_2_ to form (O_2_·)^−^ and H_2_O_2_, which then leads to the formation of the highly active, unstable and most damaging oxidant ·OH via an iron-catalyzed Haber-Weiss reaction [39, 42, 43]:

$$\text{Fe}\left(\text{II}\right)+{\text{O}}_{2}\to \text{Fe}\left(\text{III}\right)+{\left({\text{O}}_{2}\cdot \right)}^{-}$$

$$\text{Fe}\left(\text{II}\right)+{\left({\text{O}}_{2}\cdot \right)}^{-}+2{\text{H}}^{+}\to \text{Fe}\left(\text{III}\right)+{\text{H}}_{2}{\text{O}}_{2}$$

$$\text{Fe}\left(\text{II}\right)+{\text{H}}_{2}{\text{O}}_{2}\to \cdot \text{OH}+\text{O}{\text{H}}^{-}+\text{Fe}\left(\text{III}\right)$$

Nuclear factor (erythroid-derived 2)-like2 (Nrf2) is an important contributor to the reduction of oxidative stress. The main function of Nrf2 is to transcriptionally activate genes containing antioxidant response elements (ARE). Kelch-like ECH-associated protein 1 (Keap1) is the main regulator of Nrf2, and plays a central role in sensing and protecting cells against ROS. Under normal conditions, Nfr2 binds to Keap1, which promotes degradation of Nrf2 [44]. Upon exposure to ROS, Keap1 is inactivated, Nrf2 disassociates from Keap1 and becomes stabilized, and heterodimerizes with small musculoaponeurotic fibrosarcoma (Maf) proteins to drive transcription of antioxidant enzymes [45].

ARE-containing antioxidant enzymes counterbalance the harmful effects of ROS through a variety of mechanisms [46, 47]. For this study, we focus on 4 enzymes that contribute to the antioxidant response: superoxide dismutase (SOD), catalase (CAT), glutathione peroxidase (GPx), and heme oxygenase-1 (HO-1). Of these, GPx and HO-1 are directly inducible by Nrf2. Different isoforms of SOD exist in the cytosol and mitochondria, but both catalyze the dismutation of superoxide radical (O_2_·)^−^ to form hydrogen peroxide H_2_O_2_ and O_2_ [39, 42]. GPx in cytosol and mitochondria and CAT in tissue peroxisomes can reduce H_2_O_2_ to water and O_2_ to control production of the hydroxyl radical ·OH [39, 41]. The hydroxyl radical has a very short half-life of approximately 10^−9^ seconds, and is largely scavenged by endogenous and dietary ·OH scavengers (e.g. melatonin, vitamin E) whose concentration cannot be accurately predicted. These variables have therefore not been considered in our study. Besides playing a destructive role, ROS can also act as signaling molecules to promote cell proliferation, survival, apoptosis, differentiation, and migration [43, 48]. For example, ROS induce the synthesis of the inflammatory cytokine IL-6 [49, 50], and also act as signaling molecules in the EGFR oncogenic pathway.

#### Oncogenic pathways

The epidermal growth factor receptor (EGFR) regulates cell growth, differentiation, and motility through interaction with its ligand, epidermal growth factor (EGF). EGFR activation stimulates transient activation of Ras-GTP, and this eventually leads to activation of extracellular-signal-regulated kinases (ERKs) [51], which in turn results in phosphorylation and stabilization of c-Myc [52]. It has been established that c-Myc stimulates the expression of the iron regulatory protein 2 (IRP2) [53] and activates transferrin receptor 1 (TfR1) [54], which provides a link between oncogenic and iron homeostasis pathways. Ras is a small guanosine triphosphatase (GTPase), and its activity is controlled by a regulated GDP/GTP cycle. The duration of Ras activity (time spent in the GTP-bound form) and the level of activation (GTP-bound form / total Ras) are controlled by (a) the guanine nucleotide exchange factors (GEFs) that promote exchange of GDP for GTP, and (b) GTPase-activating proteins (GAPs) that stimulate the intrinsic GTPase activity of Ras to promote formation of the inactive, GDP-bound form of Ras. The activator of Ras is a GEF protein, son of sevenless (SOS), which facilitates the switch from Ras-GDP to Ras-GTP. Both SOS and Ras-GAP are recruited to phosphorylated EGFR [51, 55]. ERK phosphorylates SOS, resulting in its dissociation from growth factor receptor-bound protein 2 (Grb2) providing a negative feedback and thus limiting activation of Ras [55, 56]. Ras is also activated by IL-6 [57, 58].

## Results

Based on the known biology described in the previous section, we have constructed a network model, depicted in Fig 1. We have incorporated simplifications, as follows. Recall that HO-1 is part of the iron utilization pathway, and thus this enzyme is modeled in our network as a separate node. On the other hand SOD, CAT and GPx, which can eliminate specific reactive oxygen species, are represented as a single node, labeled *Antioxidant enzymes* (AE). Similarly, (O_2_·)^−^, H_2_O_2_ and ·OH are modeled as one species, labeled ROS in our network (Fig 1). Oncogenic pathways and reactive oxygen species (ROS) have a close and intricate relationship. Our model is not detailed enough to capture all the complexities of their interactions, but we do include many known established connections. In particular, it has been shown that activated Ras induces the production of ROS, which is required for oncogene-mediated cellular transformation and Ras dependent proliferation [59–62]. Moreover, there is a direct induction of EGFR by endogenous H_2_O_2_ and a localized generation of H_2_O_2_ by EGFR through an NADPH oxidase (Nox)-mediated process [48, 63]. Extracellular-signal regulated kinases (ERKs) and Ras are also involved in the oxidative pathway by activating Nrf2 [64–66].

The model in Fig 1 was built in a very general way, and is based primarily on the pertinent literature, including several connections derived from different cell types. For clarity, we refer to the network in Fig 1 as the *normal cell network*.

### Dynamic Model

We describe a discrete dynamic model of the network in Fig 1, based on an encoding of the regulatory logic for each node through a “logical” update rule. This type of model is qualitative, in the sense that each species can assume a finite set of states rather than quantitative concentrations of molecular species. For this study, we adopted a ternary logic, an extension of Boolean logic. Our choice of ternary logic was motivated by the fact that iron levels cannot be viewed as either ON = 1 or OFF = 0. The iron homeostasis pathway is the major focus of our study and both low and high levels of iron are detrimental, so that it is tightly controlled. Additionally, IRP2 at both low and high activity levels does alter the iron pathway [68]. With only two states it would not be clear when IRP2 operates at low activity levels, as it would be represented the same way as normally active protein. For our model to be able to differentiate between iron homeostasis (normal levels of iron) and low/high iron levels as well as activities/concentration levels of various proteins, we chose to represent each species by three levels: *low*, *normal* and *high*. In the language of logical models the state of a particular species is described by 0 if the species is low/inactive, by 1 if at normal/intermediate activity, and by 2 if high/active.

In analogy to the Boolean formalism, we can compute the future state of a species at time step *t* + 1 using the states of other species at time step *t*. Fundamental OR and AND gates for two species *X* and *Y* are defined as max{*X*, *Y*} and min{*X*, *Y*}, respectively, where *X*, *Y* ∈ {0, 1, 2}. To differentiate from the Boolean OR and AND gates, we denote these gates by Max and Min, respectively. The NOT gate (denoted here by $\bar{X}$) is defined by inverting the input, i.e., leaving 1 unchanged and inverting 0 and 2. For a concrete example, consider heme in Fig 1. It is produced through ALA synthase (ALAS1) but inhibited by HO-1. Then the logical function (update rule) that predicts how much heme is present at time *t* + 1 can be computed as follows:

$$\text{heme}\left(t+1\right)=\text{Min}\left(\text{ALAS}1\left(t\right),\bar{\text{HO}-1}\left(t\right)\right).$$

This means that, if HO-1 was 0 (low) and ALAS1 was 2 (high) at time *t*, then heme will be 2 (high) at time *t* + 1.

Based on the biological knowledge described in the previous section, we translated the interactions of the normal cell network into logical functions (see Table 1). One caveat about logical models that is not present for Boolean models is that species can change for example from a low state to a high one in one time step, skipping intermediate concentrations. This is biologically unrealistic. Thus, to address the continuity issue we have also implemented a methodology commonly used for logical models that takes into account the previous state of the regulated species (see [69] for details). For purposes of simulation, we converted the logical rules into polynomial functions to obtain a so-called *polynomial dynamical system* (PDS). A description of the construction of the PDS and the entire system can be found in the Materials and Methods section and in the supplemental file S1 PDS, respectively.

**Table 1:** Summary of all model variables and their logical update rules. An asterisk (*) in the table means that the strength of regulation for that species was adjusted based on the biology (see Materials and Methods for description and supplemental file S1 PDS).

Classification	Variable	Update Rule
Iron Homeostasis	LIP	Min(Max(TfR1, HO-1), Min($\bar{\text{Fpn}}$, $\bar{\text{Ft}}$, $\bar{\text{Mfrn}}$))
	TfR1	Max(*IRP1, IRP2, c-Myc)
	Fpn	Min(*$\bar{\text{IRP}1}$, $\bar{\text{IRP}2}$, $\bar{\text{Hep}}$)
	Ft	Min(*$\bar{\text{IRP}1}$, $\bar{\text{IRP}2}$)
	IRP1	$\bar{\text{LIP}}$
	IRP2	Max($\bar{\text{LIP}}$, c-Myc)
	Hep	IL-6
Iron Utilization	Mfrn	$\bar{\text{LIPmt}}$
	LIPmt	Min(Mfrn, $\bar{\text{Ftmt}}$, $\bar{\text{heme}}$)
	Ftmt	LIPmt
	ALAS1	Min($\bar{\text{heme}}$, LIPmt)
	heme	Min(ALAS1, $\bar{HO-1}$)
	HO-1	Max(heme, Nrf2)
Oxidative Stress Response	ROS	Min(Max(LIP, Ras, EGFR), $\bar{\text{AE}}$)
	Keap1	Min($\bar{\text{ROS}}$, *Nfr2)
	Nrf2	Max($\bar{\text{Keap}1}$, Ras, ERK)
	AE	Nrf2
Oncogenic	EGFR	ROS
	SOS	Max(EGFR, $\bar{\text{ERK}}$)
	GAPs	EGFR
	Ras	Min(Max(IL-6, SOS), $\bar{\text{GAPs}}$)
	ERK	Ras
	c-Myc	ERK
Inflammatory Cytokine	IL-6	Max($\bar{HO-1}$, ROS)

### Simulation Results

To analyze the dynamic properties of the model we simulated the entire state space and computed the basins of attraction of the system. For this purpose, we used an encoding of the model as a polynomial dynamical system, as described above, and customized scripts written in Perl and Python (see Materials and Methods section). The size of the model’s state space is 3^24^ = 282, 429, 536, 481, where 24 is the number of species in the network and 3 is the number of states (low, medium, high) per species. We employed a *synchronous* update schedule for the species in the network; all species were updated *simultaneously* based on the states of their input species at the previous time step. Each state leads to another state, eventually converging to a steady state or a limit-cycle (a set of recurring states), which are called *attractors*. A collection of initial states that lead to a particular attractor is termed the *basin of attraction*. Under this scheme, each state belongs to the basin of attraction of only one attractor: a *point* attractor (steady state) or a *cycle* attractor (limit-cycle). These attractors correspond to different *phenotypes* in the biological context and can describe various behaviors of the system such as homeostasis.

**Fig 2:**
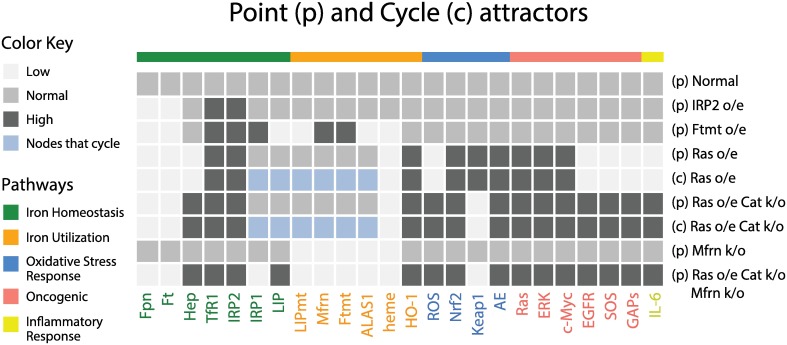
Simulation results of the intracellular iron network. Heatmap of point and cycle attractors of seven different knockout (k/o) and overexpression (o/e) models. Catalase low bioactivity (CAT k/o) is modeled by setting the AE group to zero inside the update rule for ROS (see cancer phenotype subsection for more details).

We simulated the normal cell model and also investigated the long-term behavior of this model under different conditions, namely, the effects of knockout (k/o) or overexpression (o/e) of one or more species. To simulate these experimental conditions, we set the update rule for a particular species to a constant equal to 0 or 2, respectively. In other words, regardless of the input (regulators), the species of interest will always stay at the chosen level. Our results are summarized in Fig 2, which shows all the species and their long-term behavior. Simulations were performed by exhaustively enumerating the transitions of the model on all possible 3^24^ states. The normal cell network has *no cycle* attractors and reaches a unique stable steady state (point attractor) indicating that all species are at their respective normal levels regardless of the initial starting state (Fig 2 top line of the heat map labeled *Normal*).

#### Model validation using experimental data: IRP2 overexpression only alters the iron homeostasis pathway

The iron regulatory protein IRP2 plays a central role in the regulation of the iron homeostasis pathway. It was observed that IRP2 levels are higher in breast cancer cells when compared with nonmalignant mammary epithelial cells, and it was suggested that IRP2 regulates breast tumor growth [68]. Moreover, the authors of the same study concluded that IRP2 expression levels are linked to transcriptional programs in breast cancer [68]. Thus, our initial step was to investigate whether overexpression of IRP2 in non-tumorigenic cells alters other pathways. In particular, we set the update polynomial for IRP2 to active (i.e., 2) in the normal cell model and computed the entire state space and the basin of attraction of this IRP2 overexpression model. Our simulations revealed that IRP2 overexpression leads to a single point attractor, only affects the iron homeostasis pathway, and leaves other pathways unchanged (second row in Fig 2).

**Fig 3:**
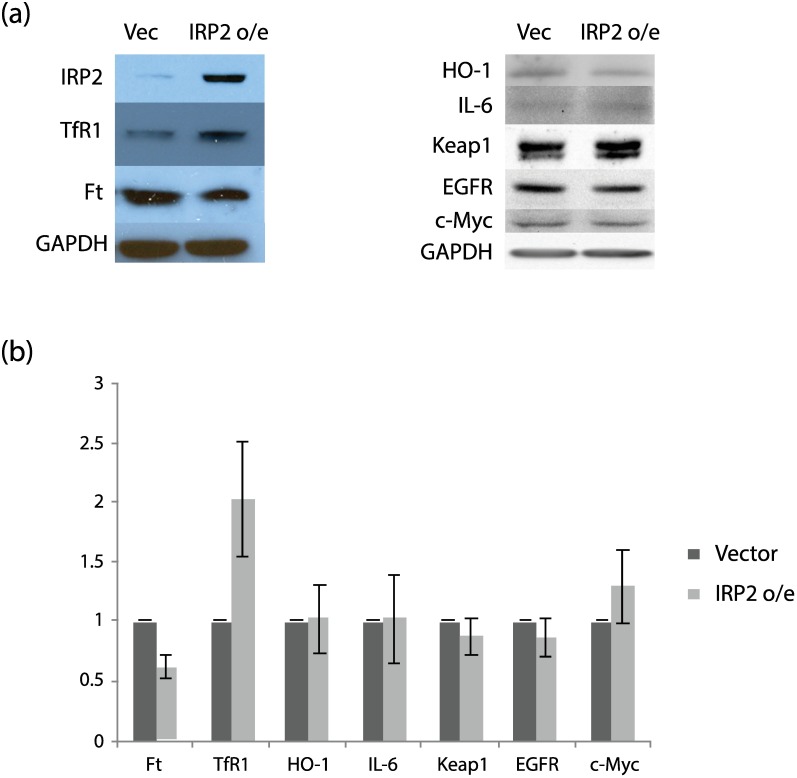
Effect of IRP2 overexpression (o/e) in MCF10A cells. (a) One representative experiment. Proteins were analyzed by Western blotting. Loading was assessed with an antibody to GAPDH. (b) Proteins in empty vector cells and IRP2 overexpressing cells. Graphs show mean and standard deviation of three separate experiments.

To test the outcome made by our model and to validate the model, we have conducted an experiment using an MCF10A non-tumorigenic immortalized human mammary breast epithelial diploid cell line [70], overexpressing IRP2 (see Materials and Methods for a detailed description of the experiment). We selected two proteins from the iron homeostasis pathway (TfR1 and Ft) and one protein from each of the other pathways in our network (HO-1, Keap1, IL-6, EGFR and c-Myc). We have found that IRP2 overexpression in MCF10A cells increases TfR1 and moderately decreases ferritin (Ft) production (Fig 3). On the other hand, there was no significant change in the levels of other proteins when IRP2 overexpressing cells were compared to MCF10A cells (Fig 3). This result agrees with our simulation results that IRP2 overexpression only alters the iron homeostasis pathway but does not have a significant effect on other pathways in the network.

#### Model validation using current literature: Mitochondrial ferritin overexpression

It was reported that overexpression of mitochondrial ferritin (Ftmt) has a dramatic effect on intracellular iron homeostasis. Specifically, Ftmt overexpression reduces both cytosolic and mitochondrial iron pools, cytosolic ferritin (Ft) and heme synthesis, and increases transferrin receptor (TFR1) levels, as well as IRP1 and IRP2 activity [71]. To test our model further, we have simulated Ftmt overexpression by setting the update rule of Ftmt to “high.” Our simulations produced a single point attractor agreeing with the experimental findings and thus again validating the model (third row in Fig 2).

#### Cancer phenotype of the iron homeostasis pathway

To investigate our hypothesis that the iron homeostasis pathway is disrupted by an oncogenic pathway in breast cancer cells, we simulated several experimental conditions to match the cancer phenotype of the iron homeostasis pathway, as suggested by the extant literature. We concentrated on two pairs of mammary epithelial cell types for which extensive experimental evidence has been previously published and for which the cancer phenotype of the iron homeostasis pathway is well defined [2, 68]. The two pairs of cell types are: (i) primary human mammary epithelial cells (HMECs) and their tumor-forming transformed variants, referred to here as R5 cells, which were generated by transduction of HMEC cells with expression vectors for SV40, hTERT and H-Ras [72], and (ii) the MCF10A non-tumorigenic immortalized human mammary epithelial diploid cell line [70], and the MCF7 breast cancer cell line, which is estrogen receptor positive (ER+), that was derived from a pleural effusion in a patient with metastatic cancer [73]. Transformed HMECs (R5 cells) contain a c-*myc* gene amplification and also a moderate increase in the level of the c-Myc protein [72]. MCF7 cells also have the c-*myc* gene amplified [74], and it has been reported that these cells have higher protein levels of Ras and, consequently, ERK activation, and higher levels of ROS than MCF10A cells [75]. Note, that Ras is frequently constitutively activated by mutations in up-stream regulators in breast cancer [76], and c-myc gene overexpression in basal-like breast cancer (∼ 50%) contributes to cancer progression and is highly associated with poor prognosis [77].

The iron homeostasis pathway in the normal network consists of seven species (see Fig 1). It was established that the levels of cytosolic LIP, TfR1, hepcidin, IRP1 and IRP2 were increased while ferroportin (Fpn) and ferritin (Ft) had decreased levels when R5 and MCF7 cell lines were compared to their non-malignant counterpart HME and MCF10A cells, respectively [2, 68]. This suggests a specific phenotype for R5 and MCF7 cell lines termed here as the *cancer phenotype of the iron homeostasis pathway* (CP-IHP). Particularly, if 1 represents individual normal levels of the seven species in HME and MCF10A cells, then the cancer phenotype can be denoted as:

(Fpn,Ft,Hep,TfR1,IRP2,IRP1,LIP)=(0,0,2,2,2,2,2). (1)

The oncogenic pathway is directly connected to two components (TfR1 and IRP2) in the iron pathway via c-Myc, which is downstream of Ras. Since Ras is highly expressed in R5 cells and is increased in MCF7 cells we began our exploration of CP-IHP by simulating the behavior of the model under the overexpression of Ras. We found that in this case the model has two attractors: a point attractor with a basin of size 74, 444, 483, 228 (26.4%) and a cycle attractor of size 207, 985, 053, 253 (73.6%) (4^th^ and 5^th^ rows in Fig 2). We note that cycle attractors depend on the mode of simulation, e.g., update schedule (synchronous vs. asynchronous).

In the Ras overexpression model, four components of the point attractor, Fpn, Ft, TfR1, and IRP2, agreed with CP-IHP as defined by Eq (1). In addition, these four species do not oscillate in the cycle attractor. Furthermore, close examination of the cycle attractor suggests that under Ras overexpression three pathways (i.e., oxidative stress, oncogenic and inflammatory) are fixed while all species that belong to the iron utilization pathway and two from the iron homeostasis pathway (LIP and IRP1) do oscillate. Even though this simulation did not produce the exact CP-IHP, it confirmed that the iron homeostasis pathway can be altered by Ras overexpression alone. Hepcidin, IRP1 and LIP did not achieve the desired levels specified by CP-IHP since (i) ROS under RAS overexpression in the model is low and thus IL-6 and, consequently, hepcidin are low, and (ii) LIP is the only regulator of IRP1 and the iron utilization pathway is still allowed to traffic iron into the mitochondria.

Breast cancer cells are frequently under persistent oxidative stress [78], and human tumor cell lines have higher levels of ROS than their non-tumorigenic versions [39]. It was found that MCF7 cells exhibited higher H_2_O_2_ levels and lower bioactivity of catalase (CAT), while the protein expression levels of CAT were higher when compared to non-malignant cells [79]. Note that CAT is part of the group termed here antioxidant enzymes (AE), and H_2_O_2_ is part of the ROS family. Thus, if at least one component of the group exhibits differential levels then we view the entire group as having higher/lower expression levels or activity. To model low bioactivity of CAT we set to zero the AE group inside the update rule for ROS. Similar to the first model, simulations of the model under the overexpression of Ras and low bioactivity of CAT revealed two attractors, with a point attractor of size 74, 461, 261, 392 (26.4%) and a cycle attractor of size 207, 968, 275, 089 (73.6%) (6^th^ and 7^th^ rows in Fig 2). This model, however, has an additional component, hepcidin, that agrees with CP-IHP, i.e., (Fpn, Ft, Hep, TfR1, IRP2) = (0, 0, 2, 2, 2).

Next, we investigated iron trafficking into the mitochondria. Recall that cytosolic iron (LIP) is transported into the mitochondria by mitoferrin (Mfrn), to be incorporated into PPIX to synthesize heme. It has been found that the level of Mfrn in MCF7 cells is lower when compared to MCF10A cells [18]. Additionally, the same study concluded that the uptake of iron ions into the mitochondria was lower in MCF7 cells, resulting in decreased mitochondrial iron accumulation (LIPmt) and a severe reduction in heme synthesis [18]. Initially, we have tested only Mfrn knockout (k/o). The simulation of the model produced a single point attractor with both LIPmt and heme at low levels, agreeing with the above finding (8^th^ row in Fig 2). Additionally, this simulation suggested that reduced levels of Mfrn alone do not affect any other pathway. Next, we have added Mfrn k/o to the latter model (Ras overexpression and low bioactivity of CAT), which resulted in the single point attractor with five out of six species from the CP-IHP achieving desired levels (9^th^ row in Fig 2):

(Fpn,Ft,Hep,TfR1,IRP2,IRP1,LIP)=(0,0,2,2,2,0,2). (2)

It is not surprising that IRP1 is low since in the network it is only down-regulated by LIP, but it is plausible that it is also regulated by other species. In addition, it has been shown that other breast cancer cell lines had variable IRP1 mRNA and protein levels [68]. Thus, IRP1 requires further investigation and careful experimentation to understand its role beyond regulation of iron metabolism in breast cancer. Even though our simulations did not produce the exact CP-IHP, we confirmed that the iron homeostasis pathway can be altered by Ras overexpression alone. Moreover, understanding and involving the iron utilization pathway seems to be the other key in differential regulation of intracellular iron homeostasis.

## Discussion

It is well-known that iron metabolism in breast epithelial cells is differentially regulated as cells transition to malignancy. Determining the causes for this altered phenotype is complicated by the complexity of iron regulation and its connection to several other processes, such as response to oxidative stress and changes in iron consumption [80], as well as crosstalk with oncogenic pathways. Integrating these different influences on the iron phenotype in normal and malignant cells can benefit greatly from a systematic approach through dynamic mathematical modeling, beyond the network approach taken in [80]. The model presented here is a first step toward a comprehensive understanding of the iron phenotype of cells as it changes in breast cancer. We have chosen to construct a qualitative model of an intracellular iron network (Fig 1) to capture its fundamental dynamic features (attractors). The main reason for our choice of modeling platform is that our current knowledge of the kinetics involved in these different processes as well as mechanisms underlying these complex reactions is very limited, so that a quantitative model, such as a system of ordinary differential equations is more challenging to construct.

We have validated our model using both experimental data and information from the literature not used in model construction. In particular, we have experimentally validated the model prediction that IRP2 overexpression in the normal cell network only alters the iron homeostasis pathway, leaving the other model components unchanged. Also, our model agrees with the current literature that overexpression of mitochondrial ferritin (Ftmt) increases both IRPs and TfR1, decreases cytosolic Ft and reduces cytosolic and mitochondrial iron pools [71]. In addition, we have shown that shutting down trafficking of iron into the mitochondria, together with Ras overexpression and Cat reduced bioactivity, does lead to the observed cancer phenotype of the iron homeostasis pathway. However, it might be possible that further refinements of the model can lead to the required phenotype by altering only the oncogenic pathway.

**Fig 4:**
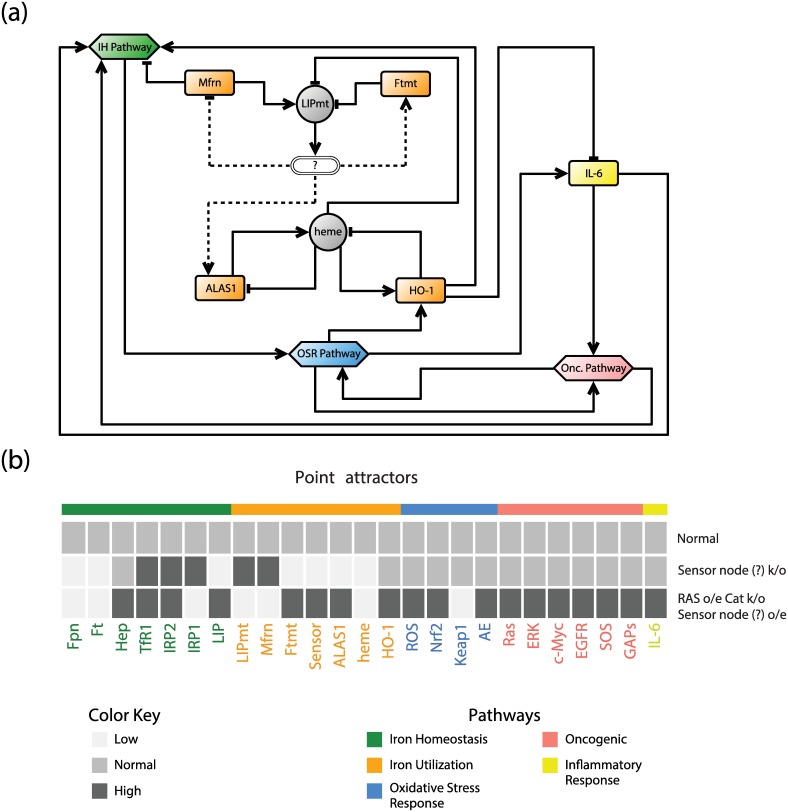
Intracellular iron network with iron-sensing node. (a) Simplified version of Fig 1 that includes a hypothesized mitochondrial iron-sensing node, which is depicted as a question mark. (b) Heat map of point attractors of three different knockout and overexpression models. (iron homeostasis (IH); oxidative stress response (OSR); oncogenic (Onc.); knockout (k/o); overexpression (o/e).)

Not all known information about the normal and cancer phenotypes can be captured by the model, however. This is likely due to the fact that some key features of this system are not represented completely, such as an iron-sensing regulator in the mitochondria and iron-sulfur cluster (ISC) synthesis. It has been suggested that frataxin, a nuclear-encoded mitochondrial protein, may act as an iron-sensing regulator and even function as a switch between heme and ISC synthesis [81–83]. At this stage, one cannot determine whether it is frataxin or some other iron sensor/regulator, but we have suggested the possibility of a mitochondrial iron-sensing node in our current model (depicted as a question mark in Fig 4(a)). This adjusted normal cell model also reaches a unique stable steady state agreeing with our model discussed in the Results section (Fig 4(b)). Additionally, we simulated two more models using the following perturbations: (i) knockout of the sensor node and (ii) overexpression of Ras and of the sensor node, and low bioactivity of CAT (2^nd^ and 3^rd^ rows in Fig 4(b)). Interestingly, the sensor node k/o model agrees with experimental data that in frataxin k/o mice heme is decreased, TfR1 is upregulated and iron uptake via Mfrn is increased, leading to cytosolic iron-deficiency and mitochondrial iron overload [84, 85]. This strongly indicates that there is a sensor/regulator, and thus further refinements of the model can provide insight into mitochondrial iron regulation and utilization, and potentially suggest new experiments that can validate new connections. The latter model produced the same cancer phenotype of the iron homeostasis pathway (see Eq (2)) and also implied that cancer cells have reduced heme biosynthesis. Furthermore, we note that the latter model allows Ftmt and ALAS1 from the iron utilization pathway to have high expression levels (compare 9^th^ row in Fig 2 to row 3 in in Fig 4(b)). While we do not have much evidence about Ftmt in cancer, there are some studies about ALAS1 in lung cancer. It was found that ALAS1 protein levels were substantially increased in non-small-cell lung cancer cells compared to normal cells [86]. This suggests the possibility to expand the cancer phenotype of the iron homeostasis pathway to the iron utilization pathway. Of course, one can simulate a model by setting various proteins to their respective observed levels, but then we gain no information about the drivers that change iron metabolism in cancer. Ideally, we would like to include other pathways implicated in breast cancer to capture different molecular subtypes of breast cancer and iron cancer phenotypes associated with them.

## Materials and Methods

### Mathematical Model

We begin by defining a set of rules that describe various relations between molecular species, from which we then build the entire model. If species X is, inducing species Y (*X* → *Y*) or species X is inhibiting species Y (*X* ⊣ *Y*) then we represent these relationships via a transition table as depicted in Table 2.

**Table 2:** Transition tables for activation and inhibition.

*X* → *Y*		*X* ⊣ *Y*	
0	0	0	2
1	1	1	1
2	2	2	0

Notice that inhibition in Table 2 is just a logical NOT gate, denoted here by $\bar{X}$. The other two fundamental gates, OR and AND, for two species *X* and *Y* regulating species *Z* (*X* → *Z* ← *Y*), are defined as max{*X*, *Y*} and min{*X*, *Y*} respectively, for *X*, *Y* ∈ {0, 1, 2}, and denoted here by Max and Min. We can express the above gates as polynomials over a finite field on three elements, $F_{3}$. If we limit the exponent of each variable in a polynomial to be less than or equal to 2, then one can show that any logical rule constructed from these three operations has a unique polynomial representation, using

$$\begin{array}{ccc} \bar{x} & = & 2+2x\\ \text{Max}\left(x,y\right) & = & x^{2}y^{2}+x^{2}y+xy^{2}+2xy+x+y\\ \text{Min}\left(x,y\right) & = & 2x^{2}y^{2}+2x^{2}y+2xy^{2}+xy. \end{array}$$ (3)

One can check that polynomials given by Eq (3) agree with definitions of fundamental gates as described in the paragraph above, e.g., max{1, 2} = 2 and Max(1, 2) = (1)^2^(2)^2^ + (1)^2^(2) + (1)(2)^2^ + 2(1)(2) + 1 + 2 = 2, where the right-hand side is computed *modulo 3*. Various adjustments to the strength of a particular regulation can be made by altering entries in the Table 2. For example, it has been suggested that IRP1, when active, contributes less to the regulation of ferritin (Ft) than IRP2 [68] (see Table 3). These tables mean that when IRP2 = 2 (active) it will inhibit Ft, whereas when IRP1 = 2 (active) it will have a lesser effect on Ft.

**Table 3:** Transition tables for IRP1 and IRP2 regulating Ft.

IRP1 ⊣ Ft		IRP2 ⊣ Ft	
0	2	0	2
1	1	1	1
2	1	2	0

Thus, we can represent regulation of Ft by IRP2 in Table 3 using Eq 3:

$$\bar{IRP2}=2+2\cdot \text{IRP}2.$$

Now, for IRP1 regulating Ft according to this new adjustment, one can also find a polynomial representing Table 3 (left table). For convenience, whenever we use an adjusted regulation we will place an asterisk (*) in front of the variable inside the logic gate.

$$*\bar{IRP2}=2+2\cdot {\left(\text{IRP}1\right)}^{2}.$$

To match current biological knowledge we have adjusted regulation of IRP1 for TfR1 and Fpn as well [68]. The transition table for Fpn is similar to Ft. For IRP1 regulating TfR1: when IRP1 = 2, then Tfr1 = 1, while, when IRP1 is 0 or 1 then TfR1 is also 0 or 1, respectively. Additionally, we modified regulation of Keap1 by Nrf2 to reflect current literature [44]. For Nrf2 regulating Keap1 we have that when Nrf2 = 0 then Keap1 = 1, while when Nrf2 is 1 or 2 then Keap1 is also 1 or 2, respectively.

To make sure that we preserve continuity (i.e., each species changes at most one unit in one time step), we are going to employ methodology as described in [69]. The underlying reasoning is that this can be accomplished by taking into account the previous state (e.g., concentration or activity) of the regulated species, in effect adding a self-regulation loop to each network node. The future value of the regulated species under continuity is computed as follows. Let *f*_*x*_*i*__ be the update function for *x*_*i*_. To ensure that each variable changes at most 1 unit, define a function *h*(*x*_*i*_, *f*_*x*_*i*__) for the future value of the variable *x*_*i*_:

$$h\left(x_{i},f_{x_{i}}\right)=\left\{\begin{array}{cc} x_{i}+1 & \text{if}\;f_{x_{i}}>x_{i}\\ x_{i} & \text{if}\;f_{x_{i}}=x_{i}\\ x_{i}-1 & \text{if}\;f_{x_{i}}<x_{i} \end{array}\right.$$ (4)

LIP, heme, and ROS do not undergo self-degradation/self-regulation and hence we do not apply continuity to these species. In order to compute final polynomials, we are going to make use of the following property of finite fields:

**Remark 0.1** *If* $h\;:\;F_{p}^{n}\to F_{p}$ *is any function then there is a polynomial* $g\;:\;F_{p}^{n}\to F_{p}$ *so that* *h*(*x*) = *g*(*x*) *for all* $x\in F_{p}^{n}$.

One can find *g* by using the following formula,

$$g\left(x\right)=\sum_{c\in F_{p}^{n}}h\left(c\right)\prod_{j}^{n}\left(1-{\left(x_{j}-c_{j}\right)}^{p-1}\right),$$ (5)

where *h*(*c*) is the update function as defined by Eq (4), *c* is a vector of input variables, and the right-hand side is computed *modulo p*.

All of these logic gates, transition tables describing different strength of regulation and continuity, are then appropriately translated into final polynomial functions over a finite field with three elements. These polynomial functions then form what is called a *polynomial dynamical system* (PDS) over a finite field. Below, we fully describe a construction of the update function for ferritin (Ft) in our network (see Fig 1). The entire PDS system can be found in the supplemental file S1 PDS.

#### Example: Ferritin (Ft)

According to our network (Fig 1), Ft has two inputs, inhibition by IRP1 and IRP2. States {0, 1, 2} for Ft will denote protein concentrations *low, medium* and *high*, respectively. Active IRP’s have high affinity for IRE’s and their binding to 5’ UTR IREs inhibits the translation of Ft. It has been suggested that active IRP2 has a greater affect on Ft, thus we will adjust the strength of each IRP as described by Table 3. The logic gate between two negated IRP’s is a Min gate:

$$f_{Ft}=\text{Min}(*\bar{IRP1,}\bar{IRP2})$$

This gate (Min) ensures that when, for example, IRP1 = 0 (inactive) and IRP2 = 2 (active), we get that Ft is inhibited by IRP2, i.e. Ft = 0 in that case, otherwise it would be 2 with a Max gate. Now we translate the above expression into a polynomial equation. First, let *x*_4_ ≔ Ft, *x*_5_ ≔ IRP1, and *x*_6_ ≔ IRP2 (this is the same assignment as we have in the supplemental file S1 PDS). The polynomial functions over a field on three elements for each transition table are:

$$*\bar{x_{5}}=2x_{5}^{2}+2\;and\;\bar{x_{6}}=2x_{6}+2$$ (6)

Using an appropriate polynomial for the Min gate as described by Eq (3), i.e., Min(*x*, *y*) = 2*x*^2^ *y*^2^ + 2*x*^2^*y* + 2*xy*^2^ + *xy*, we compute the following update function for Ft, keeping in mind that all the calculations are over $F_{3}$.

$$\begin{array}{ccc} f_{x_{4}}\left(x_{5},x_{6}\right) & = & \text{Min}\left(*\bar{x_{5}},\bar{x_{6}}\right)\\ & = & \text{Min}\left(2x_{5}^{2}+2,2x_{6}+2\right)\\ & = & 2{\left(2x_{5}^{2}+2\right)}^{2}{\left(2x_{6}+2\right)}^{2}+2{\left(2x_{5}^{2}+2\right)}^{2}\left(2x_{6}+2\right)\\ & + & 2\left(2x_{5}^{2}+2\right){\left(2x_{6}+2\right)}^{2}+\left(2x_{5}^{2}+2\right)\left(2x_{6}+2\right)\;\left(\text{simplify}\;mod\;3\;\text{and get}\right)\\ & = & x_{5}^{2}x_{6}^{2}+2x_{5}^{2}+2x_{6}+2. \end{array}$$

Now we apply the continuity process as described by Eq (4) and substitute that into Eq (5) to compute the final polynomial *f*_4_, representing an update polynomial for *x*_4_ (computations are modulo 3).

$$\begin{array}{ccc} f_{4} & = & h\left(0,f_{x_{4}}\left(0,0\right)\right)\cdot \left(1-{\left(x_{4}-0\right)}^{2}\right)\left(1-{\left(x_{5}-0\right)}^{2}\right)\left(1-{\left(x_{6}-0\right)}^{2}\right)\\ & + & h\left(1,f_{x_{4}}\left(0,0\right)\right)\cdot \left(1-{\left(x_{4}-1\right)}^{2}\right)\left(1-{\left(x_{5}-0\right)}^{2}\right)\left(1-{\left(x_{6}-0\right)}^{2}\right)\\ & + & h\left(2,f_{x_{4}}\left(0,0\right)\right)\cdot \left(1-{\left(x_{4}-2\right)}^{2}\right)\left(1-{\left(x_{5}-0\right)}^{2}\right)\left(1-{\left(x_{6}-0\right)}^{2}\right)\\ \vdots & & \\ & + & h\left(1,f_{x_{4}}\left(2,2\right)\right)\cdot \left(1-{\left(x_{4}-1\right)}^{2}\right)\left(1-{\left(x_{5}-2\right)}^{2}\right)\left(1-{\left(x_{6}-2\right)}^{2}\right)\\ & + & h\left(2,f_{x_{4}}\left(2,2\right)\right)\cdot \left(1-{\left(x_{4}-2\right)}^{2}\right)\left(1-{\left(x_{5}-2\right)}^{2}\right)\left(1-{\left(x_{6}-2\right)}^{2}\right)\\ & = & 1+x_{4}^{2}+2x_{4}^{2}x_{5}^{2}+2x_{6}+x_{4}x_{6}+2x_{4}^{2}x_{6}+x_{6}^{2}+2x_{4}x_{6}^{2}+x_{4}^{2}x_{5}^{2}x_{6}^{2}, \end{array}$$

where

$$\begin{array}{ccc} h\left(0,f_{x_{4}}\left(0,0\right)\right) & = & h\left(0,2\right)=1\\ h\left(1,f_{x_{4}}\left(0,0\right)\right) & = & h\left(1,2\right)=1\\ h\left(2,f_{x_{4}}\left(0,0\right)\right) & = & h\left(2,2\right)=2\\ \vdots & & \\ h\left(1,f_{x_{4}}\left(2,2\right)\right) & = & h\left(1,0\right)=0\\ h\left(2,f_{x_{4}}\left(2,2\right)\right) & = & h\left(2,0\right)=1. \end{array}$$

### Computational Methods

The attractors of the models were found using 2 algorithms: the attractor finder by random sampling (Algo. 1) that is written in Perl and the attractor finder by iterating over all possible states (Algo. 2) that uses a custom written Python package. The codes can be found at https://github.com/LoLab-VU/LogicalModel. Models that were used for simulations are located in the same directory under NewModels_2015_12_18 and NewModels_2015_8_17 folders. Supporting file S1 Simulations provides additional o/e and k/o simulation results using attractor finder by random sampling. The index for the order of variables is available from row 21 to row 45. After 3,000 random sampling, the basin size of the attractor is specified in the table.

The first program requires a model file, the number of states and a sampling size, which is 100,000 here. We randomly selected 100,000 states and stored the attractor states to have a broad perspective on the possible attractors of a model. It was utilized to test which overexpression and knockout models could be potential cancer models. To ensure that we know all attractors of the models of interest, we ran the second program, which requires a model file and number of states. Optional arguments include start and end states and an option to create images of attractor states. The model file is parsed and compiled into an executable function with Cython [87]. We iterated through all possible states of each model (3^*N*^), storing only the attractor states. Simulations were performed in parallel using mpi4py [88] running on large cluster computers.

**Algorithm 1** Pseudo code for attractor finder by random sampling

1: **procedure** For i in 100,000   ▹Iterate over 100,000 randomly selected states

2:  *sampled* = empty *set*

3:  *state* = changebase(random(3^*N*^))

4:  **while** *state* ∉ *sampled* **do**

5:   *sampled*.*add*(*state*)

6:   *state* = *update*(*state*)      ▹Update function is the compiled model

7:  **state.pop()**          ▹Returns the last state added to sampled

**Algorithm 2** Pseudo code for attractor finder by iterating over all possible states

1: **procedure** For i in 3^*N*^          ▹Iterate over all possible states

2:  *sampled* = empty *set*

3:  *state* = changebase(i)

4:  **while** *state* ∉ *sampled* **do**

5:   *sampled*.*add*(*state*)

6:   *state* = *update*(*state*)     ▹Update function is the compiled model

7:  **state.pop()**         ▹Returns the last state added to sampled

### Experimental Methods

MCF10A, non-tumorigenic immortalized human mammary epithelial cells were obtained from the Wake Forest University Comprehensive Cancer Center Tissue Culture Core facility. The cells were maintained in a suggested condition by ATCC.

To overexpress IRP2 in MCF10A cells, the lentiviral vector pSL2-IRP2 [68] was applied. Briefly, MCF10A cells were infected with the concentrated viral particles from pSL2-IRP2 and pLS2 empty vector (as a control). The infection efficiencies for both infections were over 90% based on GFP fluorescence in cells. The cell lysates were harvested for subsequent analysis seven days after infection.

Western blotting was performed as previously described [68]. Antibodies: GAPDH (Fitzgerald), TfR1 and c-Myc (Invitrogen), IRP2 and EGFR (Santa Cruz Biotechnology), Keap1(Cell Signaling Technology), HO-1 and IL-6 (Abcam), ferritin H ([89]).

## Supporting Information

S1 PDS Polynomial Dynamical System. The entire PDS system is coded in *Mathematica*.
(PDF)

S1 Simulations Several different simulation results of iron network model. Attractors are found using attractor finder by random sampling (Algo. 1).
(XLSX)
