# Supplementary material for: Activated Oncogenic Pathway Modifies Iron Network in Breast Epithelial Cells: A Dynamic Modeling Perspective
Source: PLoS Comput Biol. 2017 Feb 6;13(2):e1005352. doi: 10.1371/journal.pcbi.1005352 (PMC5293201; doi:10.1371/journal.pcbi.1005352)
Supplement: S1 PDS — The entire PDS system is coded in Mathematica. (PDF) [file pcbi.1005352.s001.pdf]

Supporting Document

Main article: Activated Oncogenic Pathway Modifies Iron Network in Breast Epithelial Cells: A Dynamic Modeling Perspective

Julia Chifman, Seda Arat, Zhiyong Deng, Erica Peronto, James C. Pino, Leonard A. Harris, Michael A. Kochen, Carlos F. Lopez, Steven Akman, Frank M. Torti, Suzy V. Torti, and Reinhard Laubenbacher.

This file provides computations of a PDS system over finite field $\mathbb{F}_3$. Refer to the main article for definitions and terminology.

Basic Logic Gates expressed as polynomials

x and y are elements in $\mathbb{F}_3$

```
MAX[x_, y_] := x + y + 2 x * y + x^2 * y + x * y^2 + x^2 * y^2
MIN[x_, y_] := x y + 2 x^2 y + 2 x y^2 + 2 x^2 y^2
NOT[x_] := 2 + 2 x
```

x_1 = LIP

Update function for LIP (no continuity)

```
PolynomialMod[
 PolynomialMod[MIN[MAX[x2, x8], MIN[MIN[NOT[x3], NOT[x4]], NOT[x23]]], 3] /.
  {x2^(p_) → x2^(Mod[p, 2, 1]), x8^(p_) → x8^(Mod[p, 2, 1]), x3^(p_) →
    x3^(Mod[p, 2, 1]), x4^(p_) → x4^(Mod[p, 2, 1]), x23^(p_) → x23^(Mod[p, 2, 1])}, 3]
```

```
x2 + 2 x2^2 x23 + 2 x2 x23^2 + 2 x2^2 x23^2 + 2 x2^2 x3 + x2^2 x23 x3 + 2 x2^2 x23^2 x3 + 2 x2 x3^2 +
 2 x2^2 x3^2 + 2 x2^2 x23 x3^2 + x2 x23^2 x3^2 + 2 x2^2 x4 + x2^2 x23 x4 + 2 x2^2 x23^2 x4 + x2^2 x3 x4 +
 2 x2^2 x23 x3 x4 + x2^2 x23^2 x3 x4 + 2 x2^2 x3^2 x4 + x2^2 x23 x3^2 x4 + 2 x2^2 x23^2 x3^2 x4 + 2 x2 x4^2 +
 2 x2^2 x4^2 + 2 x2^2 x23 x4^2 + x2 x23^2 x4^2 + 2 x2^2 x3 x4^2 + x2^2 x23 x3 x4^2 + 2 x2^2 x23^2 x3 x4^2 +
 x2 x3^2 x4^2 + 2 x2^2 x23 x3^2 x4^2 + 2 x2 x23^2 x3^2 x4^2 + 2 x2^2 x23^2 x3^2 x4^2 + x8 + 2 x2 x8 + x2^2 x8 +
 2 x23^2 x8 + x2 x23^2 x8 + 2 x2^2 x23^2 x8 + 2 x3^2 x8 + x2 x3^2 x8 + 2 x2^2 x3^2 x8 + x23^2 x3^2 x8 +
 2 x2 x23^2 x3^2 x8 + x2^2 x23^2 x3^2 x8 + 2 x4^2 x8 + x2 x4^2 x8 + 2 x2^2 x4^2 x8 + x23^2 x4^2 x8 +
 2 x2 x23^2 x4^2 x8 + x2^2 x23^2 x4^2 x8 + x3^2 x4^2 x8 + 2 x2 x3^2 x4^2 x8 + x2^2 x3^2 x4^2 x8 +
 2 x23^2 x3^2 x4^2 x8 + x2 x23^2 x3^2 x4^2 x8 + 2 x2^2 x23^2 x3^2 x4^2 x8 + x2 x8^2 + x2^2 x8^2 + 2 x23 x8^2 +
 x2^2 x23 x8^2 + 2 x23^2 x8^2 + 2 x2 x23^2 x8^2 + 2 x3 x8^2 + x2^2 x3 x8^2 + x23 x3 x8^2 + 2 x2^2 x23 x3 x8^2 +
 2 x23^2 x3 x8^2 + x2^2 x23^2 x3 x8^2 + 2 x3^2 x8^2 + 2 x2 x3^2 x8^2 + 2 x23 x3^2 x8^2 + x2^2 x23 x3^2 x8^2 +
 x2 x23^2 x3^2 x8^2 + x2^2 x23^2 x3^2 x8^2 + 2 x4 x8^2 + x2^2 x4 x8^2 + x23 x4 x8^2 + 2 x2^2 x23 x4 x8^2 +
 2 x23^2 x4 x8^2 + x2^2 x23^2 x4 x8^2 + x3 x4 x8^2 + 2 x2^2 x3 x4 x8^2 + 2 x23 x3 x4 x8^2 + x2^2 x23 x3 x4 x8^2 +
 x23^2 x3 x4 x8^2 + 2 x2^2 x23^2 x3 x4 x8^2 + 2 x3^2 x4 x8^2 + x2^2 x3^2 x4 x8^2 + x23 x3^2 x4 x8^2 +
 2 x2^2 x23 x3^2 x4 x8^2 + 2 x23^2 x3^2 x4 x8^2 + x2^2 x23^2 x3^2 x4 x8^2 + 2 x4^2 x8^2 + 2 x2 x4^2 x8^2 +
 2 x23 x4^2 x8^2 + x2^2 x23 x4^2 x8^2 + x2 x23^2 x4^2 x8^2 + x2^2 x23^2 x4^2 x8^2 + 2 x3 x4^2 x8^2 + x2^2 x3 x4^2 x8^2 +
 x23 x3 x4^2 x8^2 + 2 x2^2 x23 x3 x4^2 x8^2 + 2 x23^2 x3 x4^2 x8^2 + x2^2 x23^2 x3 x4^2 x8^2 + x2 x3^2 x4^2 x8^2 +
 x2^2 x3^2 x4^2 x8^2 + 2 x23 x3^2 x4^2 x8^2 + x2^2 x23 x3^2 x4^2 x8^2 + 2 x23^2 x3^2 x4^2 x8^2 + 2 x2 x23^2 x3^2 x4^2 x8^2
```

x_2 = TfR1

Rule for IRP1

```
PolynomialMod[0 * (1 - (x - 0) ^2) + 1 * (1 - (x - 1) ^2) + 1 * (1 - (x - 2) ^2), 3]
```

x^2

Define update function for TfR1

```
g2[x5_, x6_, x19_] := PolynomialMod[MAX[MAX[x5^2, x6], x19], 3]
```

Final polynomial with continuity applied

```
PolynomialMod[Expand[
  Sum[Which[c2 < g2[c5, c6, c19], c2 + 1, c2 == g2[c5, c6, c19], c2, c2 > g2[c5, c6, c19],
     c2 - 1] * (1 - (x2 - c2) ^2) * (1 - (x5 - c5) ^2) * (1 - (x6 - c6) ^2) * (1 - (x19 - c19) ^2),
   {c2, 0, 2}, {c5, 0, 2}, {c6, 0, 2}, {c19, 0, 2}]], 3]
```

$x19^2 + x2 + 2\ x19^2\ x2 + 2\ x2^2 + x19\ x2^2 + x5^2 + 2\ x19^2\ x5^2 + 2\ x2\ x5^2 + x19^2\ x2\ x5^2 + x2^2\ x5^2 + 2\ x19^2\ x2^2\ x5^2 + x2^2\ x6 + 2\ x19\ x2^2\ x6 + x19^2\ x2^2\ x6 + x6^2 + 2\ x19^2\ x6^2 + 2\ x2\ x6^2 + x19^2\ x2\ x6^2 + x19\ x2^2\ x6^2 + x19^2\ x2^2\ x6^2 + 2\ x5^2\ x6^2 + x19^2\ x5^2\ x6^2 + x2\ x5^2\ x6^2 + 2\ x19^2\ x2\ x5^2\ x6^2 + 2\ x2^2\ x5^2\ x6^2 + x19^2\ x2^2\ x5^2\ x6^2$

x_3 = Fpn

Rule for IRP1

```
PolynomialMod[2 * (1 - (x - 0) ^2) + 1 * (1 - (x - 1) ^2) + 1 * (1 - (x - 2) ^2), 3]
```

$2 + 2\ x^2$

Define update function for Fpn

```
g3[x5_, x6_, x7_] := PolynomialMod[MIN[MIN[2 + 2 * x5^2, NOT[x6]], NOT[x7]], 3]
```

Final polynomial with continuity applied

```
PolynomialMod[Expand[
  Sum[Which[c3 < g3[c5, c6, c7], c3 + 1, c3 == g3[c5, c6, c7], c3, c3 > g3[c5, c6, c7],
     c3 - 1] * (1 - (x3 - c3) ^2) * (1 - (x5 - c5) ^2) * (1 - (x6 - c6) ^2) * (1 - (x7 - c7) ^2),
   {c3, 0, 2}, {c5, 0, 2}, {c6, 0, 2}, {c7, 0, 2}]], 3]
```

$1 + x3^2 + 2\ x3^2\ x5^2 + 2\ x6 + x3\ x6 + 2\ x3^2\ x6 + x6^2 + 2\ x3\ x6^2 + x3^2\ x5^2\ x6^2 + 2\ x7 + x3\ x7 + 2\ x3^2\ x7 + x6\ x7 + 2\ x3\ x6\ x7 + x3^2\ x6\ x7 + 2\ x6^2\ x7 + x3\ x6^2\ x7 + 2\ x3^2\ x6^2\ x7 + x7^2 + 2\ x3\ x7^2 + x3^2\ x5^2\ x7^2 + 2\ x6\ x7^2 + x3\ x6\ x7^2 + 2\ x3^2\ x6\ x7^2 + x6^2\ x7^2 + 2\ x3\ x6^2\ x7^2 + 2\ x3^2\ x6^2\ x7^2 + 2\ x3^2\ x5^2\ x6^2\ x7^2$

$x_4 = \text{Ft}$

Rule for IRP1

```
PolynomialMod[2 * (1 - (x - 0) ^2) + 1 * (1 - (x - 1) ^2) + 1 * (1 - (x - 2) ^2), 3]
```

$2 + 2 x^2$

Define update function for Ft

```
g4[x5_, x6_] := PolynomialMod[MIN[2 + 2 * x5^2, NOT[x6]], 3]
```

Final polynomial with continuity applied

```
PolynomialMod[
 Expand[Sum[Which[c4 < g4[c5, c6], c4 + 1, c4 == g4[c5, c6], c4, c4 > g4[c5, c6], c4 - 1] *
     (1 - (x4 - c4) ^2) * (1 - (x5 - c5) ^2) * (1 - (x6 - c6) ^2),
    {c4, 0, 2}, {c5, 0, 2}, {c6, 0, 2}]], 3]
```

$1 + x4^2 + 2\, x4^2\, x5^2 + 2\, x6 + x4\, x6 + 2\, x4^2\, x6 + x6^2 + 2\, x4\, x6^2 + x4^2\, x5^2\, x6^2$

$x_5 = \text{IRP1}$

Define update function for IRP1

```
g5[x1_] := PolynomialMod[NOT[x1], 3]
```

Final polynomial with continuity applied

```
PolynomialMod[
 Expand[Sum[Which[c5 < g5[c1], c5 + 1, c5 == g5[c1], c5, c5 > g5[c1], c5 - 1] *
     (1 - (x5 - c5) ^2) * (1 - (x1 - c1) ^2), {c5, 0, 2}, {c1, 0, 2}]], 3]
```

$1 + 2\, x1 + x1^2 + x1\, x5 + 2\, x1^2\, x5 + x5^2 + 2\, x1\, x5^2$

$x_6 = \text{IRP2}$

Define update function for IRP2

```
g6[x1_, x19_] := PolynomialMod[MAX[NOT[x1], x19], 3]
```

Final polynomial with continuity applied

```
PolynomialMod[Expand[
   Sum[Which[c6 < g6[c1, c19], c6 + 1, c6 == g6[c1, c19], c6, c6 > g6[c1, c19], c6 - 1] *
     (1 - (x6 - c6) ^2) * (1 - (x1 - c1) ^2) * (1 - (x19 - c19) ^2),
    {c6, 0, 2}, {c1, 0, 2}, {c19, 0, 2}]], 3]
```

$1 + 2\, x1 + x1^2 + x1\, x19^2 + 2\, x1^2\, x19^2 + x1\, x6 + 2\, x1^2\, x6 + 2\, x1\, x19^2\, x6 + x1^2\, x19^2\, x6 + x6^2 + 2\, x1\, x6^2 + x1^2\, x19\, x6^2 + x1\, x19^2\, x6^2 + x1^2\, x19^2\, x6^2$

x_7 = Hep

Define update function for Hepcidin

```
g7[x15_] := PolynomialMod[x15, 3]
```

Final polynomial with continuity applied

```
PolynomialMod[
 Expand[Sum[Which[c7 < g7[c15], c7 + 1, c7 == g7[c15], c7, c7 > g7[c15], c7 - 1] *
    (1 - (x7 - c7) ^2) * (1 - (x15 - c15) ^2), {c7, 0, 2}, {c15, 0, 2}]], 3]
```

$x15^2 + x7 + 2\ x15^2\ x7 + 2\ x7^2 + x15\ x7^2$

x_8 = HO-1

Define update function for HO-1

```
g8[x10_, x13_] := PolynomialMod[MAX[x10, x13], 3]
```

Final polynomial with continuity applied

```
PolynomialMod[Expand[
  Sum[Which[c8 < g8[c10, c13], c8 + 1, c8 == g8[c10, c13], c8, c8 > g8[c10, c13], c8 - 1] *
    (1 - (x8 - c8) ^2) * (1 - (x10 - c10) ^2) * (1 - (x13 - c13) ^2),
   {c8, 0, 2}, {c10, 0, 2}, {c13, 0, 2}]], 3]
```

$x10^2 + x13^2 + 2\ x10^2\ x13^2 + x8 + 2\ x10^2\ x8 + 2\ x13^2\ x8 + x10^2\ x13^2\ x8 + 2\ x8^2 +$
$x10\ x8^2 + x13\ x8^2 + 2\ x10\ x13\ x8^2 + x10^2\ x13\ x8^2 + x10\ x13^2\ x8^2 + x10^2\ x13^2\ x8^2$

x_9 = ALAS1

Define update function for ALAS1

```
g9[x10_, x22_] := PolynomialMod[MIN[NOT[x10], x22], 3]
```

Final polynomial with continuity applied

```
PolynomialMod[Expand[
  Sum[Which[c9 < g9[c10, c22], c9 + 1, c9 == g9[c10, c22], c9, c9 > g9[c10, c22], c9 - 1] *
    (1 - (x9 - c9) ^2) * (1 - (x10 - c10) ^2) * (1 - (x22 - c22) ^2),
   {c9, 0, 2}, {c10, 0, 2}, {c22, 0, 2}]], 3]
```

$x22^2 + 2\ x10\ x22^2 + x10^2\ x22^2 + x9 + 2\ x22^2\ x9 + x10\ x22^2\ x9 +$
$2\ x10^2\ x22^2\ x9 + 2\ x9^2 + x22\ x9^2 + 2\ x10^2\ x22\ x9^2 + 2\ x10\ x22^2\ x9^2 + 2\ x10^2\ x22^2\ x9^2$

x_{10} = Heme

Update function for Heme (no continuity)

```
PolynomialMod[MIN[NOT[x8], x9], 3]
```

```
x9 + 2 x8^2 x9 + 2 x8 x9^2 + 2 x8^2 x9^2
```

x_{11} = ROS

Update function for ROS (no continuity)

```
PolynomialMod[PolynomialMod[MIN[MAX[MAX[x1, x16], x21], NOT[x12]], 3] /.
  {x1^(p_) → x1^(Mod[p, 2, 1]), x16^(p_) → x16^(Mod[p, 2, 1]),
   x21^(p_) → x21^(Mod[p, 2, 1]), x12^(p_) → x12^(Mod[p, 2, 1])}, 3]
```

```
x1 + 2 x1^2 x12 + 2 x1 x12^2 + 2 x1^2 x12^2 + x16 + 2 x1 x16 + x1^2 x16 + 2 x12^2 x16 + x1 x12^2 x16 +
 2 x1^2 x12^2 x16 + x1 x16^2 + x1^2 x16^2 + 2 x12 x16^2 + x1^2 x12 x16^2 + 2 x12^2 x16^2 + 2 x1 x12^2 x16^2 +
 x21 + 2 x1 x21 + x1^2 x21 + 2 x12^2 x21 + x1 x12^2 x21 + 2 x1^2 x12^2 x21 + 2 x16 x21 + x1 x16 x21 +
 2 x1^2 x16 x21 + x12^2 x16 x21 + 2 x1 x12^2 x16 x21 + x1^2 x12^2 x16 x21 + x16^2 x21 + 2 x1 x16^2 x21 +
 x1^2 x16^2 x21 + 2 x12^2 x16^2 x21 + x1 x12^2 x16^2 x21 + 2 x1^2 x12^2 x16^2 x21 + x1 x21^2 + x1^2 x21^2 +
 2 x12 x21^2 + x1^2 x12 x21^2 + 2 x12^2 x21^2 + 2 x1 x12^2 x21^2 + x16 x21^2 + 2 x1 x16 x21^2 +
 x1^2 x16 x21^2 + 2 x12^2 x16 x21^2 + x1 x12^2 x16 x21^2 + 2 x1^2 x12^2 x16 x21^2 + x16^2 x21^2 +
 x1 x16^2 x21^2 + x12 x16^2 x21^2 + 2 x1^2 x12 x16^2 x21^2 + 2 x1 x12^2 x16^2 x21^2 + 2 x1^2 x12^2 x16^2 x21^2
```

x_{12} = Antioxidant Enzymes (AE)

Define update function for AE

```
g12[x13_] := PolynomialMod[x13, 3]
```

Final polynomial with continuity applied

```
PolynomialMod[Expand[
  Sum[Which[c12 < g12[c13], c12 + 1, c12 == g12[c13], c12, c12 > g12[c13], c12 - 1] *
    (1 - (x12 - c12)^2) * (1 - (x13 - c13)^2), {c12, 0, 2}, {c13, 0, 2}]], 3]
```

```
x12 + 2 x12^2 + x12^2 x13 + x13^2 + 2 x12 x13^2
```

x_{13} = Nrf2

Define update function for Nrf2

```
g13[x14_, x16_, x18_] := PolynomialMod[MAX[NOT[x14], MAX[x16, x18]], 3]
```

Final polynomial with continuity applied

```
PolynomialMod[
 Expand[Sum[Which[c13 < g13[c14, c16, c18], c13 + 1, c13 == g13[c14, c16, c18],
     c13, c13 > g13[c14, c16, c18], c13 - 1] * (1 - (x13 - c13) ^2) *
    (1 - (x14 - c14) ^2) * (1 - (x16 - c16) ^2) * (1 - (x18 - c18) ^2),
   {c13, 0, 2}, {c14, 0, 2}, {c16, 0, 2}, {c18, 0, 2}]], 3]
```

$1 + x13^2 + 2\,x14 + x13\,x14 + 2\,x13^2\,x14 + x14^2 + 2\,x13\,x14^2 + x13^2\,x14^2\,x16 + x14\,x16^2 + 2\,x13\,x14\,x16^2 + x13^2\,x14\,x16^2 + 2\,x14^2\,x16^2 + x13\,x14^2\,x16^2 + x13^2\,x14^2\,x16^2 + x13^2\,x14^2\,x18 + 2\,x13^2\,x14^2\,x16\,x18 + x13^2\,x14^2\,x16^2\,x18 + x14\,x18^2 + 2\,x13\,x14\,x18^2 + x13^2\,x14\,x18^2 + 2\,x14^2\,x18^2 + x13\,x14^2\,x18^2 + x13^2\,x14^2\,x18^2 + x13^2\,x14^2\,x16\,x18^2 + 2\,x14\,x16^2\,x18^2 + x13\,x14\,x16^2\,x18^2 + 2\,x13^2\,x14\,x16^2\,x18^2 + x14^2\,x16^2\,x18^2 + 2\,x13\,x14^2\,x16^2\,x18^2$

x_{14} = Keap1

Rule for Nrf2

```
PolynomialMod[1 * (1 - (x - 0) ^2) + 1 * (1 - (x - 1) ^2) + 2 * (1 - (x - 2) ^2), 3]
```

$1 + x + 2\,x^2$

Define update function for Keap1

```
g14[x11_, x13_] := PolynomialMod[MIN[NOT[x11], 1 + x13 + 2 * x13^2], 3]
```

Final polynomial with continuity applied

```
PolynomialMod[Expand[
  Sum[Which[c14 < g14[c11, c13], c14 + 1, c14 == g14[c11, c13], c14, c14 > g14[c11, c13],
     c14 - 1] * (1 - (x14 - c14) ^2) * (1 - (x11 - c11) ^2) * (1 - (x13 - c13) ^2),
   {c14, 0, 2}, {c11, 0, 2}, {c13, 0, 2}]], 3]
```

$1 + 2\,x11 + x11^2 + x11\,x14 + 2\,x11^2\,x14 + 2\,x11\,x14^2 + x11^2\,x14^2 + x13\,x14^2 + 2\,x11^2\,x13\,x14^2 + 2\,x13^2\,x14^2 + x11^2\,x13^2\,x14^2$

x_{15} = IL-6

Define update function for IL-6

```
g15[x8_, x11_] := PolynomialMod[MAX[NOT[x8], x11], 3]
```

Final polynomial with continuity applied

```
PolynomialMod[Expand[Sum[Which[c15 < g15[c8, c11], c15 + 1,
     c15 == g15[c8, c11], c15, c15 > g15[c8, c11], c15 - 1] * (1 - (x15 - c15) ^2) *
    (1 - (x8 - c8) ^2) * (1 - (x11 - c11) ^2), {c15, 0, 2}, {c8, 0, 2}, {c11, 0, 2}]], 3]
```

$1 + x15^2 + 2\,x8 + x11^2\,x8 + x15\,x8 + 2\,x11^2\,x15\,x8 + 2\,x15^2\,x8 + x11^2\,x15^2\,x8 + x8^2 + 2\,x11^2\,x8^2 + 2\,x15\,x8^2 + x11^2\,x15\,x8^2 + x11\,x15^2\,x8^2 + x11^2\,x15^2\,x8^2$

x_{16} = Ras

Define update function for Ras

```
g16[x15_, x17_, x20_] := PolynomialMod[MIN[MAX[x15, x17], NOT[x20]], 3]
```

Final polynomial with continuity applied

```
PolynomialMod[
 Expand[Sum[Which[c16 < g16[c15, c17, c20], c16 + 1, c16 == g16[c15, c17, c20],
     c16, c16 > g16[c15, c17, c20], c16 - 1] * (1 - (x16 - c16) ^2) *
    (1 - (x15 - c15) ^2) * (1 - (x17 - c17) ^2) * (1 - (x20 - c20) ^2),
   {c16, 0, 2}, {c15, 0, 2}, {c17, 0, 2}, {c20, 0, 2}]], 3]
```

$x15^2 + x16 + 2\,x15^2\,x16 + 2\,x16^2 + x15\,x16^2 + x16^2\,x17 + 2\,x15\,x16^2\,x17 + x15^2\,x16^2\,x17 + x17^2 + 2\,x15^2\,x17^2 + 2\,x16\,x17^2 + x15^2\,x16\,x17^2 + x15\,x16^2\,x17^2 + x15^2\,x16^2\,x17^2 + 2\,x15^2\,x20 + x15^2\,x16\,x20 + 2\,x15^2\,x16^2\,x20 + 2\,x17^2\,x20 + x15^2\,x17^2\,x20 + x16\,x17^2\,x20 + 2\,x15^2\,x16\,x17^2\,x20 + 2\,x16^2\,x17^2\,x20 + x15^2\,x16^2\,x17^2\,x20 + x15^2\,x20^2 + 2\,x15^2\,x16\,x20^2 + 2\,x15\,x16^2\,x20^2 + 2\,x15^2\,x16^2\,x20^2 + 2\,x16^2\,x17\,x20^2 + x15\,x16^2\,x17\,x20^2 + 2\,x15^2\,x16^2\,x17\,x20^2 + x17^2\,x20^2 + 2\,x15^2\,x17^2\,x20^2 + 2\,x16\,x17^2\,x20^2 + x15^2\,x16\,x17^2\,x20^2 + 2\,x16^2\,x17^2\,x20^2 + 2\,x15\,x16^2\,x17^2\,x20^2$

x_{17} = SOS

Define update function for SOS

```
g17[x18_, x21_] := PolynomialMod[MAX[NOT[x18], x21], 3]
```

Final polynomial with continuity applied

```
PolynomialMod[Expand[
  Sum[Which[c17 < g17[c18, c21], c17 + 1, c17 == g17[c18, c21], c17, c17 > g17[c18, c21],
     c17 - 1] * (1 - (x17 - c17) ^2) * (1 - (x18 - c18) ^2) * (1 - (x21 - c21) ^2),
   {c17, 0, 2}, {c18, 0, 2}, {c21, 0, 2}]], 3]
```

$1 + x17^2 + 2\ x18 + x17\ x18 + 2\ x17^2\ x18 + x18^2 + 2\ x17\ x18^2 + x17^2\ x18^2\ x21 + x18\ x21^2 + 2\ x17\ x18\ x21^2 + x17^2\ x18\ x21^2 + 2\ x18^2\ x21^2 + x17\ x18^2\ x21^2 + x17^2\ x18^2\ x21^2$

x_{18} = ERK

Define update function for ERK

```
g18[x16_] := PolynomialMod[x16, 3]
```

Final polynomial with continuity applied

```
PolynomialMod[Expand[
  Sum[Which[c18 < g18[c16], c18 + 1, c18 == g18[c16], c18, c18 > g12[c16], c18 - 1] *
    (1 - (x18 - c18) ^2) * (1 - (x16 - c16) ^2), {c18, 0, 2}, {c16, 0, 2}]], 3]
```

$x16^2 + x18 + 2\ x16^2\ x18 + 2\ x18^2 + x16\ x18^2$

x_{19} = c-Myc

Define update function for c-Myc

```
g19[x18_] := PolynomialMod[x18, 3]
```

Final polynomial with continuity applied

```
PolynomialMod[Expand[
  Sum[Which[c19 < g19[c18], c19 + 1, c19 == g19[c18], c19, c19 > g19[c18], c19 - 1] *
    (1 - (x19 - c19) ^2) * (1 - (x18 - c18) ^2), {c19, 0, 2}, {c18, 0, 2}]], 3]
```

$x18^2 + x19 + 2\ x18^2\ x19 + 2\ x19^2 + x18\ x19^2$

x_{20} = GAPs

Define update function for GAPs

```
g20[x21_] := PolynomialMod[x21, 3]
```

Final polynomial with continuity applied

```
PolynomialMod[Expand[
  Sum[Which[c20 < g20[c21], c20 + 1, c20 == g20[c21], c20, c20 > g20[c21], c20 - 1] *
    (1 - (x20 - c20) ^2) * (1 - (x21 - c21) ^2), {c20, 0, 2}, {c21, 0, 2}]], 3]
```

```
x20 + 2 x20^2 + x20^2 x21 + x21^2 + 2 x20 x21^2
```

x_{21} = EGFR

Define update function for EGFR

```
g21[x11_] := PolynomialMod[x11, 3]
```

Final polynomial with continuity applied

```
PolynomialMod[Expand[
  Sum[Which[c21 < g21[c11], c21 + 1, c21 == g21[c11], c21, c21 > g21[c11], c21 - 1] *
    (1 - (x21 - c21) ^2) * (1 - (x11 - c11) ^2), {c21, 0, 2}, {c11, 0, 2}]], 3]
```

```
x11^2 + x21 + 2 x11^2 x21 + 2 x21^2 + x11 x21^2
```

x_{22} = LIPmt

Update function for LIPmt (no continuity)

```
PolynomialMod[
 PolynomialMod[MIN[x23, MIN[NOT[x10], NOT[x24]]], 3] /. {x23^(p_) → x23^(Mod[p, 2, 1]),
   x10^(p_) → x10^(Mod[p, 2, 1]), x24^(p_) → x24^(Mod[p, 2, 1])}, 3]
```

```
x23 + 2 x10^2 x23 + 2 x10 x23^2 + 2 x10^2 x23^2 + 2 x23^2 x24 + x10 x23^2 x24 +
 2 x10^2 x23^2 x24 + 2 x23 x24^2 + x10^2 x23 x24^2 + 2 x23^2 x24^2 + 2 x10 x23^2 x24^2
```

x_{23} = Mfrn

Define update function for Mfrn

```
g23[x22_] := PolynomialMod[NOT[x22], 3]
```

Final polynomial with continuity applied

```
PolynomialMod[Expand[
  Sum[Which[c23 < g23[c22], c23 + 1, c23 == g23[c22], c23, c23 > g23[c22], c23 - 1] *
    (1 - (x23 - c23) ^2) * (1 - (x22 - c22) ^2), {c23, 0, 2}, {c22, 0, 2}]], 3]
```

$1 + 2\, x22 + x22^2 + x22\, x23 + 2\, x22^2\, x23 + x23^2 + 2\, x22\, x23^2$

x_{24} = Ftmt

Define update function for Ftmt

```
g24[x22_] := PolynomialMod[x22, 3]
```

Final polynomial with continuity applied

```
PolynomialMod[Expand[
  Sum[Which[c24 < g24[c22], c24 + 1, c24 == g24[c22], c24, c24 > g24[c22], c24 - 1] *
    (1 - (x24 - c24) ^2) * (1 - (x22 - c22) ^2), {c24, 0, 2}, {c22, 0, 2}]], 3]
```

$x22^2 + x24 + 2\, x22^2\, x24 + 2\, x24^2 + x22\, x24^2$
